# ﻿Two new species of *Psen* Latreille, 1796 (Hymenoptera, Apoidea, Crabronidae) from China, with a key to *Psen* species of China

**DOI:** 10.3897/zookeys.1224.133244

**Published:** 2025-01-22

**Authors:** Yao Deng, Li Ma, Qiang Li

**Affiliations:** 1 Department of Entomology, College of Plant Protection, Yunnan Agricultural University, Kunming, Yunnan, 650201, China Yunnan Agricultural University Kunming China

**Keywords:** Crabronidae, digger wasps, key, taxonomy

## Abstract

Two new species of the genus *Psen* Latreille, 1796, namely *Psenfronistriatus***sp. nov.** and *Psenscabrosus***sp. nov.** are described and illustrated from China. A key to the Chinese species of *Psen* is also provided.

## ﻿Introduction

China encompasses 16 regions of the world’s 34 biodiversity hotspots, as identified by Conservation International (Myers et al. 2000). Over the past 8 million years, China has been profoundly shaped by geological events such as continental drift, the retreat of the ancient Mediterranean Sea, and the erosion of plateau surfaces due to the uplift of the Tibetan Plateau and the development of deep rift. These events have not only shaped its geologic history and distribution pattern but have also created unique landscape, geomorphology, microhabitat differentiation, and geographic isolation. This geologic complexity has facilitated the rapid differentiation of many biological communities, making China one of the most diverse regions in the Northern Hemisphere (Boufford 2014; Price et al. 2014; Xing and Ree 2017; Fu et al. 2024). Straddling the Palearctic and Eastern Oceanic zones in the global zoogeography, China’s diverse geomorphological patterns and climatic environments generate high environmental heterogeneity. This, in turn, supports a variety of habitats for insects in insects, contributing to an exceptionally rich biodiversity of species, with many species exhibiting macro- and trans-zonal distribution, with numerous endemics species, placing China in a significant and unique position within global zoogeography.

The genus *Psen* Latreille, 1796, belongs to the tribe Psenini in the subfamily Pemphredoninae and is the second-largest genus in the tribe. The genus was erected by Latreille with no species included, and Latreille (1802) later designated *Sphexater* Fabricius, 1794 [= *Crabroater* Olivier, 1792] as the type species of the genus. Currently, the genus *Psen* includes 95 species and 22 subspecies. These species are distributed across multiple regions: 11 species and two subspecies occur in the Palearctic, five in the Nearctic, 48 species and 17 subspecies in the Oriental, four in the Ethiopian, nine in the Neotropical, eight species and three subspecies in the Australo-Papuan, eight in each the Palearctic and Oriental, and four in each the Oriental and Australo-Papuan regions (Cameron, 1899; Turner 1912; Gussakovskij 1932; Malloch 1933; Beaumont 1937; Merisuo 1938; Tsuneki 1959, 1966, 1967, 1971, 1973, 1974, 1982, 1983; van Lith 1959, 1965, 1968, 1973, 1974, 1975, 1976, 1978; Lomholdt 1975; Bohart and Menke 1976; Kazenas 1978; Budrys 1986; Wu and Zhou 1996; Nagase 2000; Amarante 2002; Dollfuss 2004; Pulawski 2024). Of the 95 species, 26 species and six subspecies are recorded from China, comprising 27.3% of the global total, of which 20 species and one subspecies are distributed on the mainland (Yunnan, Guizhou, Sichuan, Guangdong, Guangxi, Xizang, Zhejiang, Fujian, Chongqing, Hubei, Jiangxi, Shandong, Shanxi, Henan, Beijing, Gansu, Qinghai, Inner Mongolia, Liaoning, Heilongjiang), with 11 species and five subspecies are distributed in Taiwan. Four species and one subspecies occur both in the mainland and Taiwan together (Gussakovskij 1932; Malloch 1933; Beaumont 1937; Merisuo 1938; van Lith 1968, 1974, 1975; Tsuneki 1974, 1983; Bohart and Menke 1976; Kazenas 1978; Budrys 1986; Wu and Zhou 1996; Hua 2006; Ma and Li 2006; Ma and Li 2007; Jiang et al. 2021).

The biology of *Psen* has been studied by several researchers (e.g., Girard 1879; Barth 1907; Malloch 1933; Gussakovskij 1937; Beaumont 1937, 1964; Iwata 1938; van Lith 1959, 1965, 1968; Janvier 1956; Tsuneki 1959; Steiner 1986; Woydak 1996; Kazenas 2001). Evans (1959) described the larvae of *Psen*. Members of this genus typically nests in stumps, rotten wood, hard sandy soil or mud, often with multiple nesting chambers present in decaying wood. *Psenafinis* Gussakovskii, 1937, *Psenaurifrons* Tsuneki, 1959, and *Psenbetremi* van Lith, 1959 prey on species of the family Cicadellidae; *Psenater* (Olivier, 1972), *Psencoriaceus* van Lith, 1959, *Psencurvipilosus* van Lith, 1959, *Psenerythropoda* Rohwer, 1910, *Psenrichardsi* Tsuneki, 1959, and *Psenvechti* van Lith, 1959 prey on species in the family Cercopidae; and *Psenemarginatus* van Lith, 1959 preys on Membracidae.

Many researchers have conducted taxonomic studies in China in recent decades, leading to the discovery of new species. In this study, two new species of *Psenfronistriatus* sp. nov. and *Psenscabrosus* sp. nov. collected from Yunnan, Guangdong, Shaanxi, and Inner Mongolia, China, were discovered and are described in detail, and a key to the known species in China is provided, with high-quality color photographs of the two new species. Notably, *P.fronistriatus* was first collected in Ganquan County, Yan’an, Shaanxi Province, in 1971 (as a male), and 35 years later, both a female and male were obtained from Helan Mountain, Inner Mongolia. Meanwhile, *P.scabrosus* was first collected in 2007 from Guanyin Mountain, Fogang, Guangdong Province (as a female), and was later rediscovered in the same region in 2021. These findings further demonstrate the environmental complexity of China, which not only provides diverse habitat and ecological barriers for many species but also supports a wealth of micro-ecological environments that contribute to the country’s extraordinary species diversity.

## ﻿Materials and methods

The examined specimens are deposited in Yunnan Agricultural University, Kunming, China (**YNAU**). Specimens were photographed using a stereomicroscope (Keyence VHX-S550E) equipped with a digital microscopic system. Plates were processed with Adobe Photoshop® 2020 software. For the terminology we mainly follow Bohart and Menke (1976). The abbreviations are as follows:

**HLD** head length in dorsal view (distance from the frons to the occipital margin in the middle)

**HLF** head length in frontal view (distance from the vertex to the clypeal margin in the middle)

**HW** head width (dorsal view, maximum)

**AOD** antenna-ocular distance (frontal view)

**WAS** width of antennal socket (frontal view)

**IAD** interantennal distance (frontal view)

**POD** post-ocellar distance (distance between the inner margins of the hind ocellus)

**OOD** ocellocular distance (distance between the outer margin of the hind ocellus and the nearest inner orbit)

**OCD** ocello-occipital distance (distance between the posterior margin of the hind ocellus and the occipital margin, dorsal view)

**PW** petiole width (dorsal view, in the middle)

**PL** petiole length (lateral view)

**LT I** maximum length of gastral tergum I (dorsal view)

**WT I** maximum width of gastral tergum I (dorsal view)

**HFL** maximum length of hind femur

**HTL** maximum length of hind tibia.

## ﻿Taxonomic account

### ﻿ Psen

Taxon classificationAnimaliaHymenopteraCrabronidae

Latreille, 1796

630682D2-3493-5A99-A490-3AC990B01A0A

Psen Latreille, 1796: 122 (no included species). Type species: Sphexater Fabricius, 1794 [= Crabroater Olivier, 1792], designated by Latreille 1802: 338 (first included species).
Psenus Rafinesque, 1815: 124. Emendation of Psen Latreille, 1796.
Mesopora Wesmael, 1852: 279. Type species: Psenater of Vander Linden, 1829 [= Sphexater of Panzer, 1799 = Sphexater Fabricius, 1794 = Crabroater Olivier, 1792], by monotypy.

#### Diagnosis.

The genus *Psen* can be identified as a member of the tribe Psenini Costa, 1858 within the subfamily Pemphredoninae based on Mandible bidentate apically; occipital carina joining hypostomal carina before midventral line of head; no genal process; scrobe sulus deep, hypoepimeral area raised; omaulus ending as it becomes ventral and turning a little posteriorly; forewing second recurrent vein ending in second or interstitial or third submarginal cell, hindwing M diverging before cu-a; propodeum usually coarsely reticulate posteriorly, sometimes multivariate; dorso-median area of petiole usually smooth, rarely with coarse punctures, without carinae but rarely with a posterior longitudinal groove, no conspicuous laterodorsal setae but abundant strong setae lateroventrally; male gastral sterna III and IV, or only III or IV, with marginal setae posteriorly, rarely without marginal setae, VIII an upturned pseudo-sting; female pygidial plate subtriangular, narrow or broad, sparsely or densely bristled (Bohart and Menke 1976).

### ﻿Key to the species of *Psen* Latreille, 1796 from China

**Female** (females are unknown for *P.spinitibialis* Ma & Li, 2007; *P.foveicornis* Tsuneki, 1982; *P.seriatispinosus* Ma & Li, 2006; *P.assamensis* van Lith, 1965; *P.shukuzanus* Tsuneki, 1972)

**Table:** 

1	Gastral terga with dense or sparse, long or short marginal setae posteriorly	**2**
–	Gastral terga without marginal setae posteriorly (Fig. 1A)	**7**
2	Petiole cylindrical, without carina or keel, cylindrical	**3**
–	Petiole subquadrate, lateral surface with one lateral carina on each side (Figs 1B, 3B), ventral surface without or with a keel	**5**
3	Propodeal enclosure with sturdy longitudinal rugae, propodeal pad narrow, impunctate, smooth, and shiny (Fig. 3F); gastral terga with long, straight marginal setae posteriorly; petiole with blue shine; clypeus with golden setae (Fig. 3C) (China: Taiwan)	***P.sauteri* van Lith, 1968**
–	Propodeal enclosure with slender longitudinal rugae, sometimes extending to propodeal pad (Fig. 1F); gastral terga with straight or somewhat curving, short setae, petiole without shine; clypeus with golden or silvery setae (Fig. 2C)	**4**
4	All legs largely fulvous or reddish brown; gastral terga I and II, sternum II largely reddish brown to dark brown, remainder area bright reddish brown; clypeus with golden setae (Fig. 3C) (China: Zhejiang, Sichuan)	***P.lacuniventris* Ma & Li, 2007**
–	Legs black except gray-white tibial spur, tarsus brown; gaster black; clypeus with silvery setae (Fig. 2C) (China: Taiwan)	***P.terayamai* Tsuneki, 1982**
5	Propodeal enclosure ill delimited, not impressed; second recurrent vein of forewing ending in second submarginal cell (Fig. 3A); ventral surface of petiole with a keel posteriorly, and large punctures on each side; pygidial plate with one or two rows of large punctures and setae (Fig. 3H) (China: Yunnan, Guangxi, Sichuan, Taiwan; Indonesia)	***P.lieftincki* van Lith, 1959**
–	Propodeal enclosure well delimited by triangular or lunular carina (Fig. 1F), not or shallowly impressed; second recurrent vein of forewing ending in third submarginal cell or interstitial (Fig. 1I); ventral surface of petiole without keel, impunctate or with microscopic punctures on each side; pygidial plate with five or six rows of large punctures and setae	**6**
6	Acetabular carina short, 0.7× longer than foretarsus I; gastral terga with sparse marginal setae, short and straight, silvery; gastral segments I and II, petiole ventrally bright reddish (China: Hunan, Yunnan; Nepal; India)	***P.rufoannulatus* Cameron, 1907**
–	Acetabular carina lacking; gastral terga with dense marginal setae, long, straight, golden; gastral segment I partly reddish brown to dark brown, gastral segment II and petiole black (China: Yunnan)	***P.yunnanensis* Ma & Li, 2007**
7	Petiole cylindrical, lateral surface not carinate or with slender lateral carinae, ventral surface without keel	**8**
–	Petiole subquadrate, lateral surface with slender or sturdy lateral carinae (Fig. 3B), ventral surface without or with a keel	**15**
8	Mandible much broadened	**9**
–	Mandible narrow or just somewhat broad medially or apically	**10**
9	Propodeal pad narrow, smooth, shiny (Fig. 1F); upper frons with dense, fine punctures; ocellar area, vertex with dense, midsized punctures, without groove behind hind ocelli (Fig. 1E) (China: Hubei, Zhejiang, Guangdong, Guizhou; Japan)	***P.kulingensis* van Lith, 1965**
–	Propodeal pad with dense, slender, longitudinal rugae (Fig. 3E); upper frons, ocellar area, vertex with sparse, tiny punctures, shiny, with a deep transverse groove behind hind ocelli (China: Taiwan)	***P.shirozui* Tsuneki, 1966**
10	Mandible somewhat broad medially or apically	**11**
–	Mandible narrow	**13**
11	Pygidial plate broad triangular, mat, with several rows of large punctures and setae; second recurrent vein of forewing ending in interstitial of second submarginal cell (Fig. 3A) (China: Shandong; Korea; Japan)	***P.aurifrons* Tsuneki, 1959**
–	Pygidial plate narrow triangular, smooth, shiny, with a few midsized punctures or impunctate basally; second recurrent vein of forewing ending in third submarginal cell (Fig. 1I)	**12**
12	Ocellar area, vertex slightly convex; gaster black (China: Yunnan; Nepal; India)	***P.simlensis* van Lith, 1968**
–	Ocellar area flat, vertex distinctly convex; gastral terga I–IV posteriorly, terga V–VI, and sternum III except median area bright yellowish brown, remainder area black (China: Sichuan; Indonesia)	***P.rubicunduslawkensis* van Lith, 1959**
13	Upper frons, ocellar area, vertex with very sparse, fine punctures; scutum with sparse, tiny punctures anteriorly and laterally, remainder with sparse, fine to midsized punctures; legs largely fulvous (China: Yunnan; Philippines; India; Japan)	***P.opacus* van Lith, 1959**
–	Upper frons, ocellar area, vertex with dense, small to midsized punctures; scutum with dense, fine to large punctures; legs dark brown to black largely	**14**
14	Scutum with dense, fine punctures (Fig. 1E); area between ocelli with longitudinal groove, behind hind ocelli with transverse groove; lateral surface of petiole with shallow groove and a slender lateral carina on each side (China: Yunnan, Sichuan, Taiwan; Japan; Korea; Russia)	***P.affinis* Gussakovskij, 1937**
–	Scutum with dense, midsized to large punctures; area between ocelli without groove; lateral surface of petiole without groove or carina (China: Henan, Zhejiang, Fujian, Chongqing, Yunnan, Xizang; India)	***P.fuscinervis* (Cameron, 1899)**
15	Propodeal pad with sparse or dense, long, longitudinal rugae (Fig. 3E)	**16**
–	Propodeal pad narrow or broad, impunctate, smooth, shiny (Fig. 1F)	**19**
16	Mandible narrow; interantennal tooth markedly elevated; pygidial plate broad triangular, mat, with several rows of large punctures and setae, basal 1/2 slightly convex; propodeum without bronzy shine (China: Heilongjiang, Jilin, Gansu, Beijing, Shandong, Zhejiang, Shanxi; Worldwide distribution)	***P.ater* (Olivier, 1972)**
–	Mandible much broadened, leaf-like medially and apically, subapical area with a virginal tooth; interantennal tooth moderately elevated; pygidial plate narrow triangular, coriaceous, mat or somewhat shiny, with one or two rows of punctures and setae, not convex; propodeum with bronzy shine	**17**
17	Lateral surface of petiole with slender lateral carinae, ventral surface with a slender keel, inconspicuously (China: Shanxi, Sichuan, Taiwan)	***P.bnun* Tsuneki, 1971**
–	Lateral surface of petiole with sturdy lateral carinae and deep groove, ventral surface with a strong keel	**18**
18	Free margin of clypeus with a deep, semicircle depression on each side of lateral area; scutum with dense, fine punctures anteriorly, remainder with dense, midsized to large punctures (China: Henan, Zhejiang)	***P.ussuriensis* van Lith, 1959**
–	Free margin of clypeus without depression laterally; scutum with dense, large punctures (Fig. 3C) (China: Guangdong)	***P.scabrosus* sp. nov.**
19	Propodeal pad broadly quadrate, impunctate, smooth, shiny	**20**
–	Propodeal pad narrow, impunctate, smooth, shiny (Fig. 1F)	**21**
20	Pygidial plate coriaceous and mat, with one or two rows of large punctures and setae (Fig. 1J) (China: Beijing, Shandong, Zhejiang, Fujian, Guangdong, Guangxi, Yunnan; Indonesia; India; Sri Lanka; Malay Archipelago; Japan; Nepal)	***P.nitidus* van Lith, 1959**
–	Pygidial plate smooth and shiny, with a row of large punctures and setae (Fig. 3H) (China: Hainan; Indonesia)	***P.amboinensis* van Lith, 1965**
21	Pygidial plate narrow triangular, smooth, shiny (Fig. 3H), without or with a few punctures	**22**
–	Pygidial plate elongate triangular, coriaceous, mat (Fig. 1J) or somewhat shiny, with one or two rows of fine or midsized punctures	**23**
22	Interantennal tooth moderately elevated, bluntly tooth-like; second recurrent vein of forewing ending in second submarginal cell interstitial; antenna fulvous largely (China: Sichuan; Japan)	***P.bettohattenuatus* Tsuneki, 1977**
–	Interantennal tooth slightly elevated, coniform; second recurrent vein of forewing ending in second submarginal cell (Fig. 3A); antenna beneath brown (China: Taiwan; Korea; Russia; Japan)	***P.koreanusformosensis* Tsuneki, 1965**
23	Mandible narrow; pronotal collar with anterior-lateral corners, not forming tooth (China: Taiwan)	***P.alishanus* Tsuneki, 1967**
–	Mandible somewhat broad medially or apically; pronotal collar without anterior-lateral corner	**24**
24	Lateral surface of petiole with a sturdy lateral carina on each side, ventral surface with a sturdy keel (China: Jilin, Inner Mongolia, Qinghai, Xizang, Sichuan, Yunnan, Taiwan; Korea; Russia; Japan)	***P.seminitidus* van Lith, 1965**
–	Lateral surface of petiole with a slender lateral carina on each side and a deep groove, ventral surface without keel	**25**
25	Upper frons with dense, fine punctures, ocellar area, vertex with sparse, fine punctures; second recurrent vein of forewing ending in second submarginal cell; antenna beneath largely, gastral tergum I laterally, femur apically, tibia, tarsus reddish brown; head, thorax with bronzy shine (China: Taiwan)	***P.tanoi* Tsuneki, 1967**
–	Upper frons with dense, fine to midsized punctures and slender, longitudinal rugae (Fig. 1C), ocellar area, vertex with dense, midsized to large punctures (Fig. 1E); second recurrent vein of forewing ending in third submarginal cell (Fig. 1I); antenna, gaster black, leg largely black; head, thorax without bronzy shine (Fig. 1B) (China: Inner Mongolia, Shaanxi, Yunnan)	***P.fronistriatus* sp. nov.**

**Male** (males are unknown for *P.amboinensis* van Lith, 1965; *P.opacus* van Lith, 1959; *P.terayamai* Tsuneki, 1982; *P.lacuniventris* Ma & Li, 2007; *P.sauteri* van Lith, 1968; *P.scabrosus* sp. nov.)

**Table:** 

1	Gastral sterna III and IV without marginal setae posteriorly (Fig. 2B) (China: Inner Mongolia, Shaanxi, Yunnan)	***P.fronistriatus* sp. nov.**
–	Gastral sterna III and IV with marginal setae posteriorly	**2**
2	Only gastral sternum III or IV with marginal setae posteriorly	**3**
–	Gastral sterna III and IV with marginal setae posteriorly	**6**
3	Gastral sternum III with marginal setae posteriorly, dark brown, dense, somewhat long and straight; acetabular carina lacking, mesosternum with two or three sturdy, long, longitudinal carinae on each side of ventral median carina (China: Sichuan; Indonesia)	***P.rubicunduslawkensis* van Lith, 1959**
–	Gastral sternum IV with marginal setae posteriorly, fulvous or dark brown, short and straight; acetabular carina lacking or short, mesosternum without longitudinal carina	**4**
4	Mandible narrow, petiole cylindrical, lateral surface not carinate or with weak lateral carina on each side (China: Taiwan)	***P.alishanus* Tsuneki, 1967**
–	Mandible somewhat broad medially, petiole subquadrate (Fig. 2B), lateral surface with a sturdy lateral carina on each side	**5**
5	Ventral surface of petiole without keel; upper frons, ocellar area, vertex with dense or sparse, tiny punctures, ocellar area flat; antennal joints III–XII beneath with tubercles (China: Sichuan, Taiwan; Japan)	***P.bettohattenugius* Tsuneki, 1977**
–	Ventral surface of petiole with a sturdy keel; upper frons with dense, fine punctures and longitudinal rugae, ocellar area, vertex with dense, fine punctures, markedly convex; antennal joints V–VI or V–VII beneath with linear carinae (China: Jilin, Inner Mongolia, Qinghai, Xizang, Sichuan, Yunnan, Taiwan; Korea; Russia; Japan)	***P seminitidus* van Lith, 1965**
6	Gastral terga with dense or sparse, long or short marginal setae posteriorly	**7**
–	Gastral terga without marginal setae posteriorly (Fig. 2A)	**10**
7	Mandible much broadened, tooth leaf-like, inner margin with a small tooth on median area and 1/3 of apex; interantennal tooth long, nail-like, sharp (Fig. 2C); antenna beneath without tyloids; petiole strongly curving to blunt angle basally, lateral surface with a median longitudinal carina medially and posteriorly, and a pair lateral carina on each side, ventral surface with a keel and large punctures (China: Yunnan, Guangxi, Sichuan, Taiwan; Indonesia)	***P.lieftincki* van Lith, 1959**
–	Mandible narrow, inner margin with a tooth subapically; interantennal tooth small tooth-like, somewhat sharp; antenna beneath without tyloids or concave; petiole slightly curving, lateral surface not carinate or with a pair lateral carina on each side, ventral surface without or with a keel, impunctate	**8**
8	Gastral terga with sparse, short, straight, silvery marginal setae posteriorly; gastral segments I and II laterally bright reddish brown (China: Hunan, Yunnan; Nepal; India)	***P.rufoannulatus* Cameron, 1907**
–	Gastral terga with dense, long, curving or straight, golden or yellowish marginal setae posteriorly; gastral tergum I largely or wholly reddish brown, remainder black	**9**
9	Ventral surface of petiole with a keel; second recurrent vein of forewing ending in second submarginal cell interstitial; antennal joints VI–XIII beneath with oval tubercles, black, smooth, shiny (China: Zhejiang)	***P.spinitibialis* Ma & Li, 2007**
–	Ventral surface of petiole without keel; second recurrent vein of forewing ending in third submarginal cell (Fig. 2A); antennal joints VI–XI beneath with elliptic tubercles (China: Zhejiang)	***P.yunnanensis* Ma & Li, 2007**
10	Propodeal pad with longitudinal rugae or reticulation (Fig. 3E)	**11**
–	Propodeal pad narrow or broad, impunctate, smooth, shiny (Fig. 2F)	**18**
11	Midtarsus with angular or spinous produced (Fig. 4A–E); mandible narrow or somewhat broad medially and apically	**12**
–	Midtarsus not deformed (Fig. 4F, G); mandible much broadened	**15**
12	Petiole cylindrical, lateral surface without carina	**13**
–	Petiole subquadrate, lateral surface with a longitudinal carina on each side (Fig. 2B)	**14**
13	Half apex of mandible broad; mid basitarsus with auriform prominence (Fig. 4D, E); second recurrent vein of forewing ending in second submarginal cell interstitial (Fig. 3A); antennal joints VI–XIII beneath with elliptic concave (China: Shandong; Korea; Japan)	***P.aurifrons* Tsuneki, 1959**
–	Mandible narrow; each of midtarsus markedly produced posteriorly (Fig. 4C); second recurrent vein of forewing ending in third submarginal cell (Fig. 2A); antennal joints V–XIII beneath with tyloids, on joint V small, elliptic, on V–XIII large oval concave (China: Zhejiang; Guangxi; Taiwan)	***P.foveicornis* Tsuneki, 1982**
14	Inner margin of mid basitarsus with a row of 6 nail-shaped thorns basally and a long spine on 1/3 of base (Fig. 4A, B); antennal joints III–XIII beneath with tyloids (China: Guizhou)	***P.seriatispinosus* Ma & Li, 2006**
–	Mid basitarsus with an angular prominence medially and posteriorly; joints VI–XIII beneath with tyloids (China: Heilongjiang, Jilin, Gansu, Beijing, Shandong, Zhejiang, Shanxi; worldwide distribution)	***P.ater* (Olivier, 1972)**
15	Petiole cylindrical; free margin of clypeus with broad, shallow emargination (China: Taiwan)	***P.shirozui* Tsuneki, 1966**
–	Petiole subquadrate (Fig. 2B); free margin of clypeus with deep, semicircular emargination	**16**
16	Lateral surface of petiole with upper edge only, 1/2 apex area with dense, midsized punctures, ventral surface without keel; interantennal tooth blunt apically (China: Zhejiang; India)	***P.assamensis* van Lith, 1965**
–	Lateral surface of petiole with a pair lateral carina on each side, without or with deep groove medially, ventral surface with a strong keel; interantennal tooth tooth-like, sharp apically	**17**
17	Free margin of clypeus without depression; second recurrent vein of forewing ending in third submarginal cell (Fig. 2A) or interstitial; upper frons, ocellar area, vertex with sparse, tiny punctures; scutum with sparse, fine punctures (China: Shanxi; Sichuan; Taiwan)	***P.bnun* Tsuneki, 1971**
–	Free margin of clypeus with a deep semicircle depression on each side; second recurrent vein of forewing ending in second submarginal cell interstitial (Fig. 3A); upper frons, ocellar area, vertex with sparse, fine punctures; scutum with dense, fine punctures anteriorly, remainder with dense, midsized to large punctures (China: Henan, Zhejiang; Russia; Sweden; Japan; Korea)	***P.ussuriensis* van Lith, 1959**
18	Propodeal pad broadly quadrate, impunctate, smooth, shiny; acetabular carina much longer (China: Beijing, Shandong, Zhejiang, Fujian, Guangdong, Guangxi, Yunan; Indonesia; India; Sri Lanka; Malay Archipelago; Japan; Nepal)	***P.nitidus* Tsuneki, 1966**
–	Propodeal pad narrow or somewhat broad, impunctate, smooth, shiny (Fig. 2F); acetabular carina lacking, or short, or somewhat long	**19**
19	Mandible broad or at least apical 1/2 somewhat broad	**20**
–	Mandible narrow	**23**
20	Petiole subquadrate (Fig. 2B), lateral surface with a pair lateral carina on each side, without or with deep groove	**21**
–	Petiole cylindrical, lateral surface without carina or groove	**22**
21	Setae on head, thorax, leg golden; upper frons with dense, fine punctures, ocellar area, vertex with sparse, fine punctures (China: Taiwan)	***P.tanoi* Tsuneki, 1967**
–	Setae on head, thorax, leg silvery; upper frons, ocellar area, vertex with sparse, large punctures (China: Taiwan; Korea; Russia; Japan)	***P.koreanusformosensis* Tsuneki, 1965**
22	Scutum with sparse, tiny to fine punctures; ocellar area, vertex with sparse, tiny to fine punctures; antennal joints V–VIII beneath with linear carinae (China: Yunnan; India; Nepal)	***P.simlensis* van Lith, 1968**
–	Scutum with dense, large punctures; ocellar area, vertex with dense, midsized punctures; antennal joints V–VIII beneath with wide cylindrical tyloids and III and XII beneath with linear carinae (China: Hubei, Zhejiang, Guangdong, Guizhou; Japan)	***P.kulingensis* van Lith, 1965**
23	Petiole subquadrate (Fig. 2B), having a broad furrow on each side that is margined on both sides by carinae (China: Taiwan)	***P.shukuzanus* Tsuneki, 1972**
–	Petiole cylindrical, lateral surface without furrow and lateral carina or with a slender lateral carina on each side or with a shallow groove	24
24	Acetabular carina lacking; antennal joints III–XI beneath with tyloids, on joints III–X long tubercles, on joint XI short carina (China: Sichuan, Yunnan, Taiwan; Japan; Korea; Russia)	***P.affinis* Gussakovskii, 1937**
–	Acetabular carina short, 0.3× longer than foretarsus I; antennal joints IV–XIII beneath with tyloids (China: Henan; Zhejiang; Fujian; Chongqing; Yunnan; Xizang; India)	***P.fuscinervis* (Cameron, 1899)**

### ﻿ Psen fronistriatus sp. nov.

Taxon classificationAnimaliaHymenopteraCrabronidae

5AD221E3-67E8-581F-8CB9-A7B66F4A008A

https://zoobank.org/B1E624A9-0218-46B3-996B-BB83F7EA355C

Figs 1A–J , 2A-L

#### Type material.

***Holotype*.** China • ♀; Inner Mongolia, Helan Mountain; 38°57'45"N, 105°51'8"E; 24.VII.2006; 1833 m elev.; collected by Ming LUO. ***Paratypes***: China • 1♂; same data as holotype; China • 1♀; Yunnan Province, Gaoligong Mountain, Dulong River Tunnel; 27°50'56"N, 98°28'3"E; 15.VII–2.VIII.2020; 2824 m elev.; collected by Lang YI; China • 1♂; Shaanxi Province, Yan’an City, Ganquan County, Liulimao; 36°10'3"N, 109°21'26"E; 5.VII.1971; 1077 m elev.; collected by Jikun Yang. All types deposited in YNAU.

#### Diagnosis.

The new species is similar to *Psenseminitidus* van Lith, 1965, but differs from it and other congeners by the following characteristics (characters of *P.seminitidus* in parentheses): 1) the posterior surface of the propodeum lacks an oblique longitudinal ridge (the posterior surface of the propodeum has weakly oblique longitudinal carinae); 2) flagellomeres VII–X have linear tyloids beneath in the male (flagellomeres V–VI or V–VII beneath have linear tyloids); 3) the female POD: OOD: OCD = 12: 17: 21 (the female POD: OOD: OCD = 12: 13: 15); 4) the female PL: PW: LT I: WT I: HFL: HTL = 80: 14: 52: 64: 71: 88 (the female PL: PW: LT I: WT I: HFL: HTL = 69: 11: 41: 50: 60: 70); 5) the ocellus and vertex areas feature dense, midsized to large punctures (the ocellus and vertex area feature dense, fine punctures); 6) the scutum has dense, fine punctures anteriorly, dense and midsized to large punctures medially and posteriorly (the scutum has dense, fine punctures); 7) the petiole lacks a median longitudinal keel ventrally in the female (the petiole has median longitudinal keel); 8) the second recurrent vein ends in the third or interstitial submarginal cell (the second recurrent vein ends in the second submarginal cell).

#### Description.

**Figure 1.**
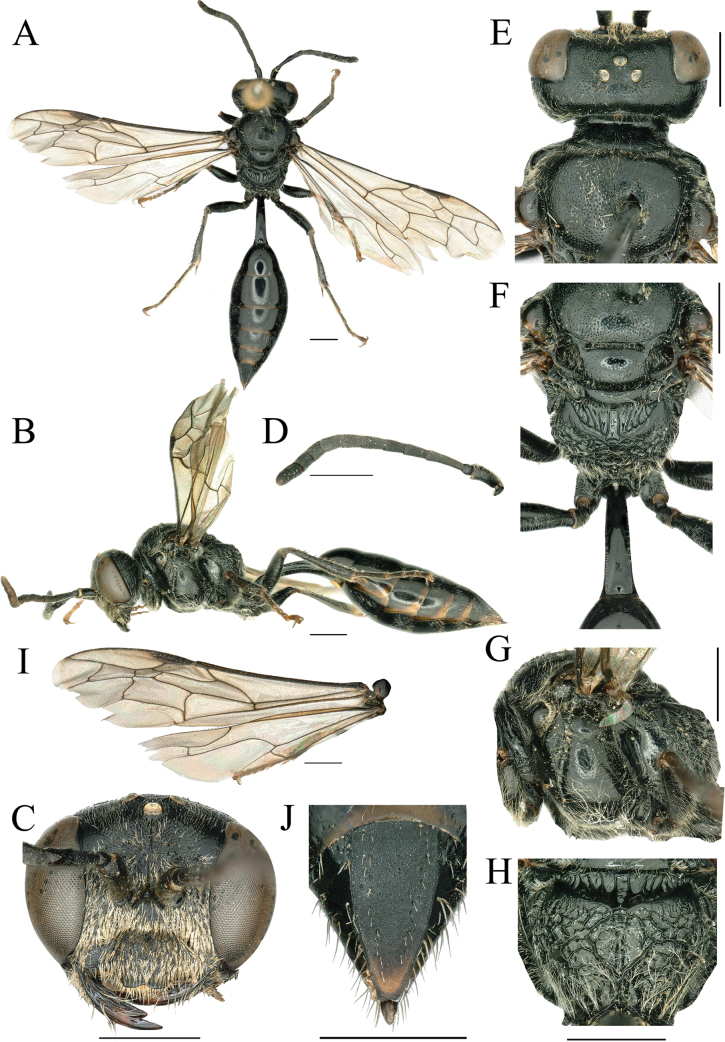
*Psenfronistriatus* sp. nov., holotype ♀ **A** habitus, dorsal view **B** habitus, lateral view **C** head, frontal view **D** antennae **E** head, pronotum and scutum, dorsal view **F** scutellum, metanotum and propodeum, dorsal view **G** thorax, lateral view **H** propodeum, posterior view **I** left wing **J** pygidial plate, dorsal view. Scale bars: 1 mm.

**Female.** Body length 11.0–11.8 mm. Black; mandible and pygidial area apically reddish brown; fore and mid tarsi dark brown. Appressed setae on clypeus golden or silvery, vertex, scutum, scutellum, and metanotum with long, dense, pale yellow pubescence. Setae on mesopleuron, legs, propodeum, and petiole mid length and silvery (Fig. 1A, B).

***Head*.** In frontal view, clypeus with dense, fine punctures except margin, mid portion prominent with arch shallow emargination medially, basal 1/2 of clypeal disk moderately convex. HW: HLF: AOD: WAS: IAD = 102: 78: 10: 8: 14. Mandible bidentate apically, broad, blunt; width basally: medially: apically = 18: 10: 3. Interantennal tooth conspicuous, high, apex obtuse or slightly acute; frontal carina weak and reaching interantennal tooth around median ocellus (Fig. 1C). Scape of antennae slightly bent, relative lengths of joints III–XII = 24–26, 16–17, 15–16, 15, 13–14, 12–13, 11–13, 11–12, 11–12, 16–17; joint III ~ 3.4–4.3× as long as wide apically, joint IV with 2.0–2.4×, joint XII with 1.8–1.9× (Fig. 1D). Frons shiny with dense, fine to midsized punctures, and below anterior ocellus with weak longitudinal ridges (Fig. 1C). In dorsal view, ocellus and vertex area shiny, with dense, midsized to large punctures, interspaces larger than frons; ocellar area not raised, behind postocelli with shallow sulcus, vertex behind postocelli region not raised, occipital carina without longitudinal ridge (Fig. 1B, E). HW: HLD: POD: OOD: OCD = 102: 36: 12: 17: 21.

***Thorax*.** Scutum with dense, fine punctures anteriorly, dense, midsized to large punctures medially and posteriorly, interspaces 1–2× as wide as diameter of puncture. Admedian lines and notauluses weak, nearly parallel (Fig. 1F). Scutellum with dense, fine punctures, metanotum with sparse, microscopic punctures (Fig. 1F). Propleuron with five or six short oblique striae, epicnemial areas shiny, with sparse, microscopic punctures, omaulus ending as it becomes ventral and below normally curved backwards. Mesopleura shiny with sparse, microscopic punctures, posteriorly without longitudinal striae (Fig. 1G). Mesosternum without acetabular carina, with slightly strong, longitudinal medioventral carina, medially with one or two transverse carinae. Enclosed area of propodeum depressed, bordered by a narrow horizontal area which is distinctly separated from back of propodeum, horizontal area slightly wider on either side of sulcus, laterally with some sturdy oblique longitudinal carinae; propodeal pad with a smooth area, slightly wider on sides and narrower in middle (Fig. 1F). Posterior surface of propodeum with sturdy reticulation, medially with deep sulcus reaching enclosed area, upper of sulcus with three or four transverse carinae (Fig. 1H). In profile, dorsal surface of propodeum with posterior surface nearly obtuse angle, upper lateral surface of propodeum with oblique, short rugae (Fig. 1G). Second recurrent vein ending in third or interstitial submarginal cell (Fig. 1I). Hind tibia with a row of long, thick, brownish thorns on outer surface (Fig. 1B).

***Gaster*.**PL: PW: LT I: WT I: HFL: HTL = 80: 14: 52: 64: 71: 88. Petiole nearly quadrate in cross section, slightly bent upwards basally, slightly widened backwards, width apically 2.2× basally, dorsally completely smooth (Fig. 1B, F). Lateral side with two slender longitudinal carinae and deeply depressed (Fig. 1B); ventrally without median longitudinal keel. Gaster shiny, terga with sparse microscopic punctures. Pygidial area elongate-triangular, coriaceous, 1.8–2.1× as long as wide basally, laterally one or two rows of coarse, midsized punctures and stiff bristles, apex truncate, slightly concave in middle, basally not convex (Fig. 1J). Sterna smooth.

**Figure 2.**
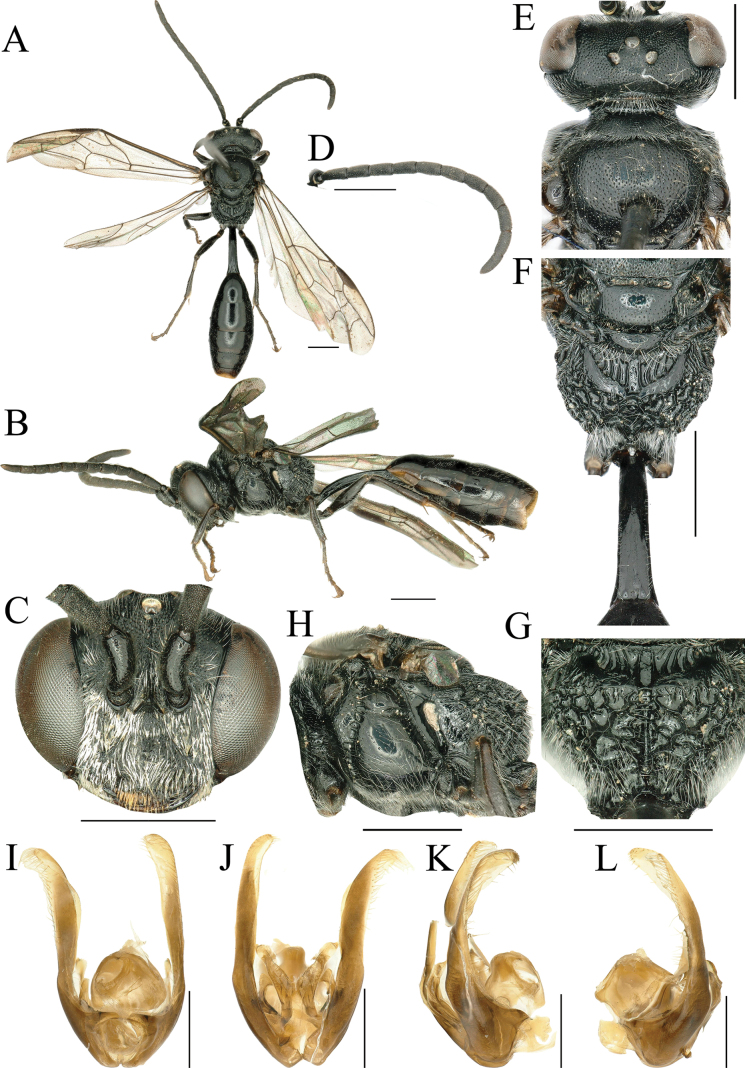
*Psenfronistriatus* sp. nov. ♂ **A** habitus, dorsal view **B** habitus, lateral view **C** head, frontal view **D** antennae **E** head, pronotum and scutum, dorsal view **F** scutellum, metanotum and propodeum, dorsal view **G** propodeum, posterior view **H** thorax, lateral view **I** male genitalia, dorsal view **J** male genitalia, ventral view **K, L** male genitalia, lateral view. Scale bars: 1 mm.

**Male.** Similar to female, but body slender, smaller, body length 9.0–11.0 mm (Fig. 2A, B). Mandible, fore and mid tibiae, and tarsi dark brown. Vertex and scutum with long, dense, palely yellow pubescence (Fig. 2C). Gastral sterna III and IV without fasciculate setae on hind margin (Fig. 2B). Clypeus mid prominent portion with arch shallow emargination in middle, two triangular protections in both sides; partially covering labrum. Frons shiny with dense, fine to midsized punctures, and below anterior ocellus with indistinct longitudinal ridges (Fig. 2C). Ocellar area slightly raised (Fig. 2A). Antennae slenderer than female, pedicel partially concealed within scape (Fig. 2A); flagellomeres VII–X beneath with linear tyloids; relative lengths of joints III–XIII = 21–24, 18–20, 17–20, 17–20, 17–19, 16–18, 15–16, 17, 16, 15–17, 20–21; joint III ~ 2.6–3.0× as long as wide apically, joint IV 2.1–2.5×, joint XII 1.6–2.0× (Fig. 2D). In frontal view, HW: HLF: AOD: WAS: IAD = 81: 61: 7: 8: 10; dorsal view, HW: HLD: POD: OOD: OCD = 81: 32: 11: 15: 16 (Fig. 2C). Hind tibia without long, thick, brownish thorns on outer surface (Fig. 2B). Petiole ventrally with median longitudinal keel medially and posteriorly, PL: PW: LT I: WT I: HFL: HTL = 70: 11: 43: 46: 53: 60. Genitalia large, yellowish brown, gonostyle slender and long, apical portion with inner (or dorsal) 1/2 turned into a semitransparent membrane, outer (or ventral) margin and apex provided with a fringe of sparse long setae (Fig. 2I–L). Volsella divided into two branches medially, dorsal and ventral, each roundly curved and united with each other again at base of apical elongate body, cuspis flattened, hollowed ventrally, with apex gently rounded and turned ventrally, slightly produced on inner apical area.

#### Distribution.

China (Inner Mongolia, Shaanxi, Yunnan).

#### Etymology.

The specific name is derived from two Latin words: *froni* - (= frons) and -*striatus* (= striate), referring to the upper frons weakly striate.

### ﻿ Psen scabrosus sp. nov.

Taxon classificationAnimaliaHymenopteraCrabronidae

43DC1125-1742-577E-B17E-F4D8A5E103FD

https://zoobank.org/F238C85C-45D6-478A-B351-E8E595FEAF02

Fig. 3A–H

#### Type material.

***Holotype*.** China • ♀; Guangdong Province, Ruyuan County Nanling National Nature Reserve; 24°56'15"N, 113°0'40"E; 26.VI–28. IX.2021; 1278 m elev.; collected by Institute of Zoology, Guangdong Academy of Sciences. ***Paratype***: China • 1♀; Guangdong Province, Fogan County, Guanyin Mountain; 23°58'13"N, 113°33'49"E; 15–16.IX.2007; 184 m elev.; collected by Zaifu XU. All types deposited in YNAU.

#### Diagnosis.

The new species is similar to *Psenleclercqi* van Lith, 1974, but differs from it and other congeners by the following characteristics (characters of *P.leclercqi* in parentheses): 1) free margin of the clypeus has three teeth, middle tooth small, lateral teeth large (free margin of the clypeus has two arch-shaped teeth); 2) the frons has coarse, midsized to large punctures, which gradually increase in size from the lower frons to the mid-ocellus (the frons up to ocelli is densely striate-punctate, interstices shining, very narrow margin along the oculi with finer and sparser punctures); 3) the vertex behind the postocellus distinctly raised (not distinctly raised); 4) the mid ocellus postero-laterally has reticulate punctures with coarse interstices (with fine, sparse punctures, interstices shiny); 5) the hind tibia has a row of long, thick, brownish thorns on the outer surface only (with row of short thick thorns and thin, long, white spines); 6) the scutellum has dense, large punctures, the diameter of punctures is 2–3× as the width of the interspaces, although medially the puncture diameter as wide as interspaces (the scutellum is somewhat striate-punctate, interstices larger than punctures); 7) antennae dark brown, yellowish brown apically, while segments III–VII reddish beneath (antennae black but underside of scape and of segments II, III, and XII are reddish brown); 8) the thorax is black (the pronotum dorsally and upper part of foreside, pronotal tubercles, anterior corners of scutum, and upper 2/3 of anterior plate of the mesepisternum are reddish brown).

#### Description.

**Figure 3.**
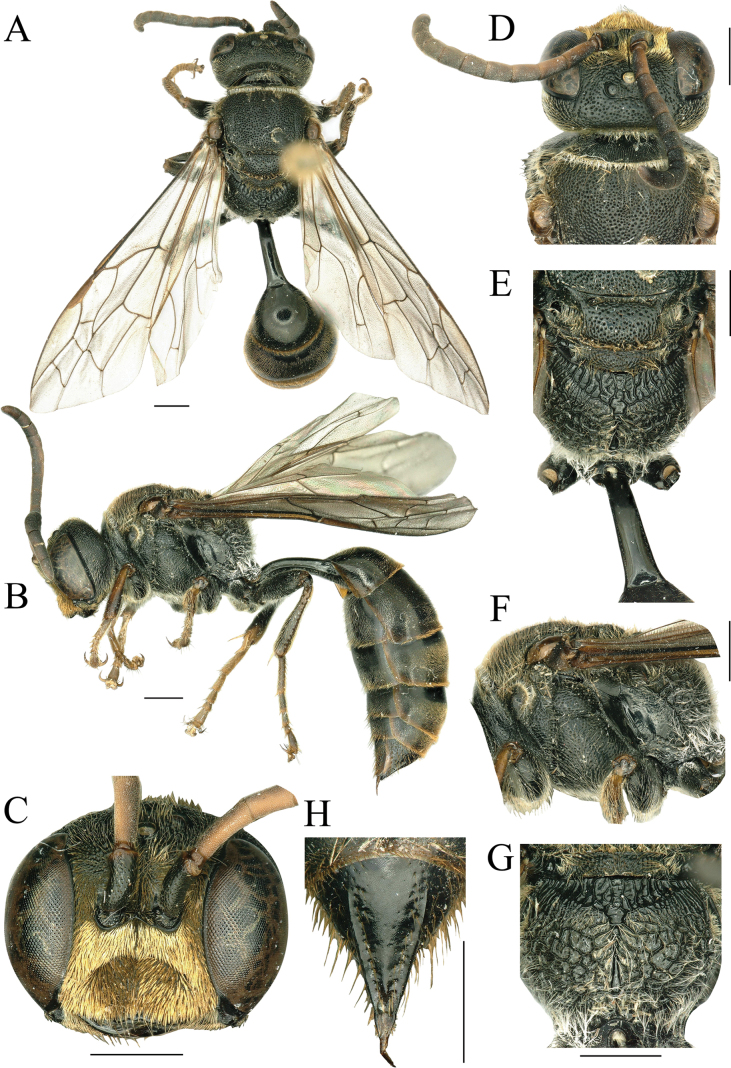
*Psenscabrosus* sp. nov., holotype ♀ **A** habitus, dorsal view **B** habitus, lateral view **C** head, frontal view **D** head, pronotum and scutum, dorsal view **E** scutellum, metanotum and propodeum, dorsal view **F** thorax, lateral view **G** propodeum, posterior view **H** pygidial plate, dorsal view. Scale bars: 1 mm.

**Female.** Body length 13.0–13.6 mm. Black (Fig. 3A, B); mandible apically reddish brown; palpi and antennae apically yellowish brown, antennae dark brown but joints III–VII reddish beneath (Fig. 3C, D). Fore and mid tibiae and tarsi reddish brown with subsequent parts yellowish brown and inner parts black; tarsi, tegulae, veins of wings, and stigma dark brown; margin of gastral terga yellowish brown (Fig. 3A, B). Appressed setae on clypeus golden, frons with less appressed golden pubescence and long erect setae. Long setae on vertex, occiput, collar, posterior margin of pronotum, scutum, scutellum, and metanotum golden (Fig. 3C–E). Mid-length setae on mesopleuron, legs, and propodeum palely yellow, prospectus and ventral side of petiole silvery; gaster with very dense, short, golden pubescence (Fig. 3B).

**Figure 4.**
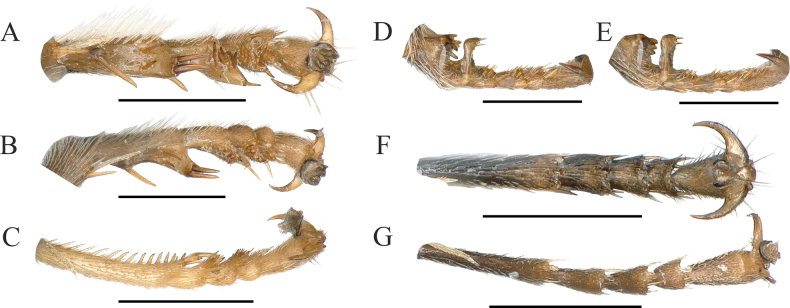
Comparison of midtarsus features of selected species of *Psen***A***P.ater* (Olivier, 1792), ventral view **B***P.ater* (Olivier, 1792), lateral view **C***P.foveicornis* Tsuneki, 1982, lateral view **D***P.shirozui* Tsuneki, 1966, lateral view **E***P.shirozui* Tsuneki, 1966, lateral view **F***P.bnun* Tsuneki, 1971, ventral view **G***P.simlensis* van Lith, 1968, ventral view. Scale bars: 0.5 mm.

***Head*.** In frontal view, clypeus with shiny margin, impunctate, free margin sinuate, with three teeth medially, middle tooth small, lateral teeth large; basal 1/2 of clypeal disk moderately convex (Fig. 3C). HW: HLF: AOD: WAS: IAD = 128: 100: 9: 12: 15. Mandible bidentate apically, broad, blunt; width basally: medially: apically = 26: 15: 5. Carina ending below antennae in low triangular tooth, connected with inner side of antennal sclerites by slender carinae, interantennal tooth distinctly high, apex straight or obtuse; frontal carina distinct and reaching interantennal tooth around median ocellus (Fig. 3C). Scape of antennae slightly bent, relative lengths of joints III–XII = 29, 21, 18, 18, 16, 16, 15, 15, 16, 23; joint III ~ 2.9× as long as wide apically, joint IV with 1.9×, joint 12 with 2.1×. Frons with coarse, midsized to large punctures, diameter of puncture 2–3× as wide as interspaces; lower 2/3 of frons slightly shiny, punctures gradually increase in size from lower frons to mid ocellus (Fig. 3C). In dorsal view, mid ocellus postero-laterally with reticulate punctures, interstices coarse; ocellar area with dense, shallow, large punctures, diameter of puncture approximately as wide as interspaces or slightly more (Fig. 3D). Vertex behind ocellus with deep, large punctures, posteriorly coarser and somewhat striate-punctate, diameter of puncture 2–3× as wide as interspaces, interspaces of vertex slightly larger than frons, barely shiny (Fig. 3D). Ocellus area not raised, behind postocellus with deep sulcus, vertex behind postocellus region distinctly raised (Fig. 3B, D). Occiput with sparse, fine punctures, occipital carina without longitudinal ridge (Fig. 3B, D). HW: HLD: POD: OOD: OCD = 128: 48: 18: 17: 36.

***Thorax*.** Scutum densely and coarsely rugose-punctate, diameter of punctures 2× as wide as interspaces, punctures on both sides arranged in longitudinal trend, interspaces slightly shiny medially; anterior 1/2 of admedian lines slightly expended, posteriorly parallel, parapsidal lines and notauluses nearly parallel (Fig. 3D). Scutellum with dense, large punctures, diameter of puncture 2–3× as wide as interspaces, but diameter of punctures medially as wide as interspaces. Metanotum with dense, oblique carinae, striate-punctate (Fig. 3E). Propleuron with dense, short striae, epicnemial areas densely and finely punctate, omaulus ending as it becomes ventral and below normally curved backwards (Fig. 3F). Mesopleura with dense, midsized to large punctures, interspaces smooth and as wide as diameter of puncture, puncture becoming smaller from top down, upper part of posterior margin of mesopleura with long striae more striking, lower part with fine punctures; subalar area with dense, large punctures, diameter of puncture 2.5× as wide as interspaces, hypo-epimeral area densely, largely striate-punctate (Fig. 3F). Metapleura shiny, with some upper transverse rugae on posterior 1/2 (Fig. 3F). Mesosternum without acetabular carina, with strong, longitudinal medioventral carina; medially with three or four strong transverse carinae. Propodeal enclosure forming a broad triangular shape, enclosed area depressed, shiny, laterally with some longitudinal carinae, medially with irregular carinae; propodeal pad with dense, slender, oblique longitudinal rugae; posterior surface of propodeum with irregular reticulate ridges reaching enclosed area (Fig. 3G). In profile, dorsal surface of propodeum together with posterior surface nearly arc-shaped, lateral surface of propodeum with oblique, short rugae and fine punctures (Fig. 3F). Second submarginal cell receiving first recurrent vein at approximately medially; second recurrent vein ending in second submarginal cell (Fig. 3A). Femora heavy, hind tibia with a row of long, thick, brownish thorns on outer surface (Fig. 3B).

***Gaster*.**PL: PW: LT I: WT I: HFL: HTL = 80: 17: 77: 87: 82: 70. Petiole nearly quadrate in cross section, slightly bent upwards basally, widened backwards, width apically 1.8× basally, dorsally completely smooth (Fig. 3B, E). Lateral side with two slender longitudinal carinae, deeply depressed medially (Fig. 3B); ventrally with an indistinct, blunt, median longitudinal keel, two sides with dense, fine punctures. Gastral terga I and II with sparse, microscopic punctures, interspaces 2× as wide as diameter of puncture; terga III and IV with dense, fine punctures medially and posteriorly, interspaces as wide as diameter of puncture, but basally 1/3 of tergum III and basally 1/4 of tergum IV smooth, impunctate. Pygidial area elongate-triangular, polished, 1.7× as long as wide basally, laterally with one or two rows of coarse punctures and stiff bristles, apex truncate, slightly concave medially (Fig. 3H). Sterna wholly with sparse, fine punctures except sternum II with dense, fine to midsized punctures.

**Male.** Unknown.

#### Distribution.

China (Guangdong).

#### Etymology.

The specific name from Latin word: *scabrosus* (= *scabrous*), referring to the head and thorax with large, scabrous punctures.

## Supplementary Material

XML Treatment for Psen

XML Treatment for Psen fronistriatus

XML Treatment for Psen scabrosus

## ﻿Additional information

### Author ORCIDs

Yao Deng https://orcid.org/0000-0002-0754-4647

Li Ma https://orcid.org/0000-0002-3436-1387

Qiang Li https://orcid.org/0000-0001-5950-8843

### Data availability

All of the data that support the findings of this study are available in the main text.
